# *Baccharis trimera* (Less.) DC Exhibits an Anti-Adipogenic Effect by Inhibiting the Expression of Proteins Involved in Adipocyte Differentiation

**DOI:** 10.3390/molecules22060972

**Published:** 2017-06-12

**Authors:** Daniele de Souza Marinho do Nascimento, Ruth Medeiros Oliveira, Rafael Barros Gomes Camara, Dayanne Lopes Gomes, Jessika Fernanda Santiago Monte, Mariana Santana Santos Pereira Costa, Júlia Moraes Fernandes, Silvana Maria Zucolotto Langassner, Hugo Alexandre Oliveira Rocha

**Affiliations:** 1 Programa de Pós-graduação em Ciencias da Saúde, Centro de Ciências da Saúde, Universidade Federal do Rio Grande do Norte-UFRN, R. Gen. Gustavo Cordeiro de Farias, s/n, Natal-RN 59012-570, Brazil; danysmari@yahoo.com.br (D.d.S.M.d.N.); dayanne_gomes@hotmail.com (D.L.G.); szucolotto@hotmail.com (S.M.Z.L.); 2 Departamento de Bioquimica, Centro de Biociencias, Universidade Federal do Rio Grande do Norte-UFRN, Av. Salgado Filho 3000, Natal-RN 59078-970, Brazil; rmo_85@hotmail.com (R.M.O.); rafael_bgc@yahoo.com.br (R.B.G.C.); jessika.monte@gmail.com (J.F.S.M.); mariana.costa@ifrn.edu.br (M.S.S.P.C.); 3 Instituto Federal de Educação, Ciência a Tecnologia do Rio Grande do Norte (IFRN), Rodovia RN-288, s/n-Nova Caicó, Caicó-RN 59300-000, Brazil; 4 Escola Multicampi de Ciências Médicas do Rio Grande do Norte -EMCM/RN, Universidade Federal do Rio Grande do Norte-UFRN, Av. Cel. Martiniano, 354, Caicó-RN 59300-000, Brazil; 5 Instituto Federal de Educação, Ciência a Tecnologia do Rio Grande do Norte (IFRN), Rua das margaridas, 300-Conjunto COHAB, Macau-RN 59500-000, Brazil; 6 Departamento de Farmácia, Centro de Ciências da Saúde, Universidade Federal do Rio Grande do Norte-UFRN, Rua General Gustavo Cordeiro de Farias, s/n, Natal-RN 59012-570, Brazil; fernandesjm@outlook.com

**Keywords:** adipogenesis, antioxidant, chlorogenic acid

## Abstract

*Baccharis trimera* (Less.) DC (gorse) is a plant popularly used for the treatment of obesity. In this study, we prepared three *B. trimera* extracts aqueous extract (AE), decoction (AE-D), and methanol extract (ME) and investigated their antioxidant effects in six different tests and their anti-adipogenic effect in 3T3-L1 cells. The extracts showed a dose-dependent antioxidant activity in all tests. AE was the most potent antioxidant in copper and ferric ion chelation assays, whereas AE-D was the most potent in superoxide and hydroxyl radical scavenging assays, reducing power assay, and total antioxidant capacity analysis. Only ME showed a cytotoxic effect against 3T3-L1 cells. Lipid accumulation decreased in 3T3-L1 adipocytes in the presence of AE and AE-D extracts (0.5 to 1.0 mg/mL). In addition, the extracts dramatically attenuated the levels of adipogenic transcriptional factors, including CCAAT enhancer-binding protein α (C/EBPα), CCAAT enhancer-binding protein β (C/EBPβ), and gamma receptors by peroxisome proliferators (PPARγ), during adipogenesis. AE-D (1.0 mg/mL) caused an approximately 90% reduction in the levels of these molecules. We propose that *B. trimera* has an anti-adipogenic effect and could be used in the development of functional foods.

## 1. Introduction

Obesity is considered an important health issue since it is a risk factor for the development of a number of chronic diseases such as cardiovascular disease (primarily heart disease and stroke), diabetes, skeletal muscle disorders, and some cancers such as endometrial, breast, and colon [1]. Human obesity is defined as a BMI (body mass index) equal to or greater than 30 kg/m^2^. A person with a BMI of 25 and 30 kg/m^2^ is considered overweight. Moreover, according to the World Health Organization, between 1980 and 2014, the obesity index worldwide more than doubled. Worldwide, in 2014, approximately 39% of adults (aged 18 years or older) were overweight and about 13% of the adult population was obese [1].

The development of adipose tissue involves both hyperplasia (increased number of cells) and hypertrophy (increase in cell size). Hyperplasia is related to the proliferation and differentiation of pre-adipocytes (adipogenesis), while hypertrophy is the result of an excessive accumulation of triglycerides in existing adipocytes [2].

Much of the knowledge about the molecular mechanisms and signals involved in adipogenesis was acquired through studies using the 3T3-L1 cell line [3]. The differentiation of these cells comprises precisely controlled sequential stages that include cell cycle arrest and clonal expansion and differentiation (first and second stages of transcription factor activation phase) through the activation of hundreds of previously silenced genes [4]. The exposure of confluent pre-cultured adipocytes (3T3-L1) to differentiation inducers leads to early events represented by the expression of CCAAT enhancer-binding proteins (C/EBPs), C/EBP-β, and C/EBP-δ [4,5]. Subsequently, the cells resume their cell cycle, undergo clonal expansion, and enter a terminal differentiation process through the activation of gamma receptors by peroxisome proliferators (PPARγ) and C/EBP-α, both key regulators in the adipogenic process [5].

Few drugs have been approved by the U.S. Food and Drug Administration (FDA) for the treatment of obesity, and use of these has been associated with the onset of side effects, including the development of cardiovascular diseases [6]. Therefore, studies in various parts of the world have focused on the discovery of new sources that have anti-obesity and anti-adipogenic properties.

Several oxidants, such as hydrogen peroxide, are involved in the development of obesity. These oxidants act as signaling messengers inducing phosphorylation, oxidation, and dimerization related to the expression of C/EBP-α and PPARγ [7]. Thus, several compounds have anti-adipogenic activity because they prevent the increase in oxidant levels [8].

*Baccharis trimera* (Less.) DC is traditionally known in Brazil as “carqueja” and all parts of the plant are popularly used to produce a tonic that is consumed for body weight loss [9].

The antimicrobial [10], anti-inflammatory, analgesic [11], and hypoglycemic [12] effects of *B. trimera* have been reported. In addition, high-fat diet-induced obese rats showed both decreased weight and less serum cholesterol after treatment with the methanol extract of *B. trimera* than did the rats that did not receive the *B. trimera* extract [13]. Corroborating these findings, in vitro tests showed that *B. trimera* methanol extract inhibits pancreatic lipase and α- and β-glucosidases [14] whereas *B. trimera* aqueous extract inhibits only glucosidases [15]. *B. trimera* aqueous extract also showed antioxidant activity in the DPPH test [16] and the total radical-trapping antioxidant parameter (TRAP) test [17] but there are no data on *B. trimera* aqueous extract evaluations using other antioxidant tests. Moreover, there are no data about the anti-adipogenic effect of *B. trimera*. Thus, this study was performed to examine the antioxidant activity of *B. trimera* extract, as well as the effect of *B. trimera* extracts on adipogenic differentiation of 3T3-L1 cells.

## 2. Results

### 2.1. Antioxidant Tests

We tested the aqueous extract (AE), methanolic extract (ME) and decoction (AE-D) of *B. trimera* for antioxidant activity. The decoction, which is subsequently used to produce a tonic, is the version most commonly used as a folk remedy, and, for this reason, we investigated its antioxidant properties.

**Figure 1:**
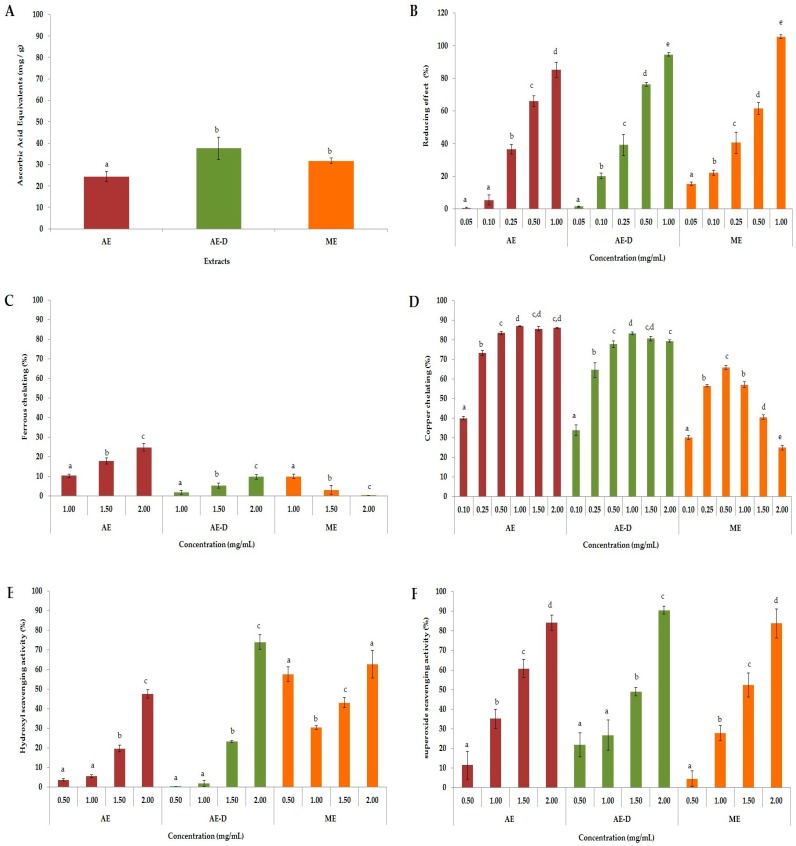
Antioxidant activities of AE (aqueous extract), AE-D (aqueous extract from decoction) and ME (methanol extract): (**A**) total antioxidant capacity; (**B**) reducing power; (**C**) ferrous chelating; (**D**) copper chelating; (**E**) hydroxyl radical scavenging; and (**F**) superoxide radical scavenging. Letters a,b,c,d represent the presence of significant difference between different concentration of the same extract as determined using one-way analyses of variance (ANOVA) followed by the Student’s *t*-test (*p* < 0.05).

As shown in Figure 1A, AE was the least effective extract, with an antioxidant activity that corresponds to 25.2 mg of ascorbic acid equivalent, while AE-D and ME exhibited 35.1 and 33.0 mg of ascorbic acid equivalent, respectively.

The reducing power assay was used to assess the ability of the sample to donate electrons, with the results shown in Figure 1B. All extracts showed a dose-dependent effect. Again, AE extract was less efficient than AE-D and ME, since only these two extracts showed nearly 100% activity at a high concentration (1.0 mg/mL).

All three extracts presented ferric chelating activity (Figure 1C), but, in this case, AE was the most potent extract (~25% activity), whereas the activity of AE-D and ME did not exceed 15%. Overall, the ferric chelating activity of the three extracts was very low compared to their cupric chelating activity. As shown in Figure 1D, AE and AE-D extracts exhibited a marked and dose-dependent cupric chelating activity, with approximately 86% and 83% of chelation, respectively. The maximal chelating activity of ME was 65% (0.5 mg/mL), but, with increasing concentration, this value decreased.

The results of the hydroxyl radical scavenging assay (Figure 1E) showed that the 0.5 mg/mL dose of ME showed an activity of ~60%, but this effect did not increase at higher doses. In contrast, AE and AE-D showed a dose-dependent effect, reaching saturation around 50% and 70%, respectively. With regard to the superoxide ion scavenging ability of *B. trimera*, we observed that all extracts showed a dose-dependent effect (Figure 1F), reaching saturation at approximately 90%.

### 2.2. Determination of the Amount of Protein, Sugar and Phenolic Compounds in the Extracts

The amount of protein, sugar, and phenolic compounds in the three extracts is shown in Table 1. There were fewer sugar and phenolic compounds in ME than in AE, although ME possessed the largest amount of proteins. AE and AE-D showed similar results, suggesting that decoction did not change the amount of protein, sugar, or phenolic compounds that are extracted with water.

### 2.3. Antiproliferative Assay

**Figure 2:**
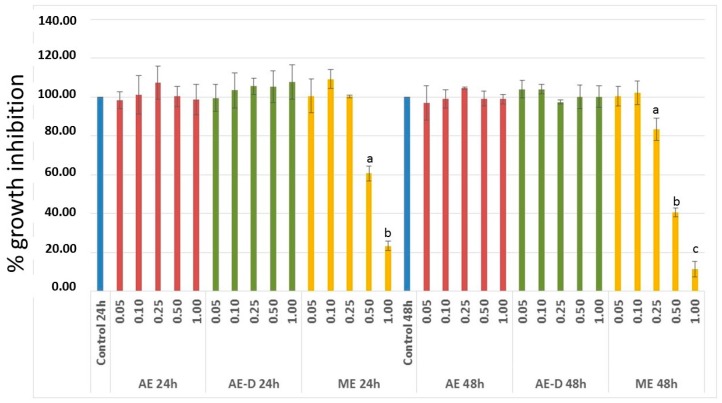
The effect of aqueous extract (AE), decoction extract (AE-D), and methanol extract (ME) on 3T3-L1 cell proliferation. 3T3-L1 cell proliferation was carried out in the presence or absence of extracts (0, 0.05, 0.1, 0.25, 0.5, and 1 mg/mL). The rate of cell proliferation inhibition was determined using BrdU. The results are expressed as mean ± SD of four determinations. Letters a,b,c, indicate significantly differences between different concentrations and the control according to one way analysis of variance (ANOVA) followed by the Student’s *t*-test (*p* < 0.05).

In order to investigate the anti-proliferative effect of the *B. trimera* extracts, 3T3-L1 cells were treated with different concentrations of the three extracts and cellular viability was assessed via BrdU assay. As shown in Figure 2, treatment with AE and AE-D (0.05–1.00 mg/mL) for 24 or 48 h did not affect the proliferation of 3T3-L1. In contrast, treatment with ME did affect 3T3-L1 cell proliferation. After 24 h, ME 0.5 mg/mL and 1.0 mg/mL decreased the proliferation of 3T3-L1 by approximately 40% and 75%, respectively. After 48 h, this inhibition was greater than that previously observed and ME 0.25 mg/mL also inhibited 3T3-L1 proliferation. Due to this result, ME was not used in the following assays.

### 2.4. Effect of B. trimera Extracts on Intracellular Lipid Accumulation

**Figure 3:**
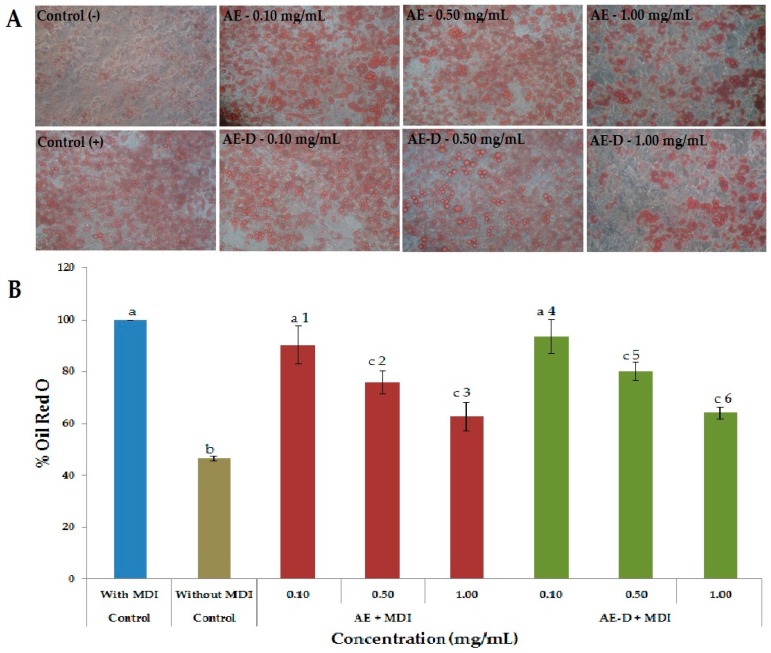
Adipocytes stained with the dye Oil Red O after 15 days after the onset of differentiation. (**A**) Intracellular lipid accumulation in 3T3-L1 cells treated with adipogenic medium (MDI) and different concentrations of AE or AE-D. After 15 days of treatment the cells were stained with oil Red O, observed with a microscope and photographed (20× magnification). (**B**) *Baccharis trimera* extracts inhibited the differentiation of 3T3-L1 preadipocytes into mature adipocytes. Oil Red O dye was used to stain the adipocytes 15 days after treatment with the extracts. Stained oil droplets were dissolved with isopropanol and evaluated by spectrophotometric analysis at 510 nm. The optical density in cells treated only with MDI was taken as 100% of relative lipid content. Values are expressed as mean ± standard deviation (*n* = 3). Letters a,b,c indicate significant differences between different concentrations with the control. Numbers 1,2,3,4,5,6 indicate significantly differences between different concentrations of same extract according to one-way analysis of variance (ANOVA) followed by the Student’s *t*-test (*p* < 0.05).

The effect of AE and AE-D on adipogenesis was assessed using 3T3-L1 cells in the presence of adipogenic medium (MDI: dexamethasone, IBMX, insulin, and fetal bovine serum) with the addition of various concentrations of AE or AE-D. The size and number of lipid droplets in 3T3-L1 adipocytes after AE and AE-D treatment were visualized using microscopy. After approximately three days of incubation in the presence of MDI, 3T3-L1 cells started to exhibit the morphology of adipocytes, including intracellular accumulation of fat droplets (Data not shown). After 15 days, the cells were stained with Oil Red O (ORO). Undifferentiated 3T3-L1 (negative control) contained fewer lipid droplets than treated cells (Figure 3A). In contrast, the number of droplets was greater in mature 3T3-L1 adipocytes (positive control). The number and size of lipid droplets decreased in mature 3T3-L1 adipocytes treated with 0.5 and 1.0 mg/mL AE or AE-D, whereas the extracts at a concentration of 0.1 mg/mL did not affect these parameters.

The levels of lipid accumulation into 3T3-L1 cells after AE and AE-D treatment were quantified using ORO staining. As shown in Figure 3B, the amount of intracellular ORO extracted from undifferentiated 3T3-L1 cells corresponded to only 50% the amount found in mature adipocytes (control, defined as 100% fat droplet content). Moreover, extracts at a low concentration (0.1 mg/mL) did not decrease the ORO amount extracted from cells. However, the level of ORO decreased significantly after treatment with 0.5 and 1.0 mg/mL of either extract (Figure 3B).

### 2.5. Determination of Free Glycerol in 3T3-L1 Cells Medium

**Figure 4:**
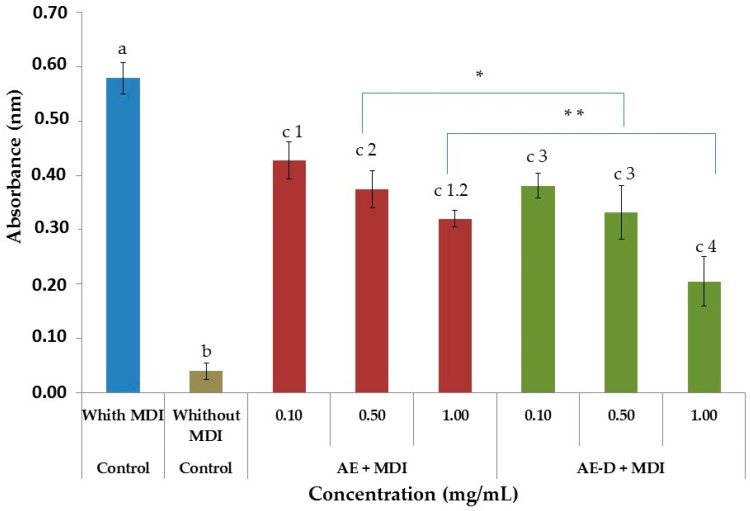
Induction of hydrolysis of intracellular lipids by *Baccharis trimera* aqueous extract (AE) and aqueous extract with decoction (AE-D). The induction of intracellular lipids hydrolysis was determined by quantification of glycerol released into the culture medium. An aliquot of the culture medium after the last change of medium was used to quantify free glycerol. Values are expressed as mean ± standard deviation (*n* = 3). Letters a,b,c indicate significant differences between different concentrations with the control. Numbers 1,2,3,4 indicate significant differences between different concentrations of same extract. * and ** indicate significant differences between AE 0.5 mg/mL and AE-D 0.5 mg/mL and between AE 1.0 mg/mL and AE-D 1.0 mg/mL according to one-way analysis of variance (ANOVA) followed by the Student’s *t*-test (*p* < 0.05).

3T3-L1 cells were seeded into plates and induced to differentiate into mature adipocytes in the presence or absence (positive control) of extracts, as described above. After 15 days, the amount of free glycerol in the culture medium was measured. A very low level of glycerol was present in the medium of undifferentiated 3T3-L1 cells, and increased approximately six times in the medium of mature 3T3-L1 adipocytes (Figure 4). In contrast, the effect of glycerol decreased in the presence of extracts in a dose-dependent manner.

### 2.6. B. trimera Extracts Affect the Levels of C/EBPα, C/EBPβ and PPARγ

**Figure 5:**
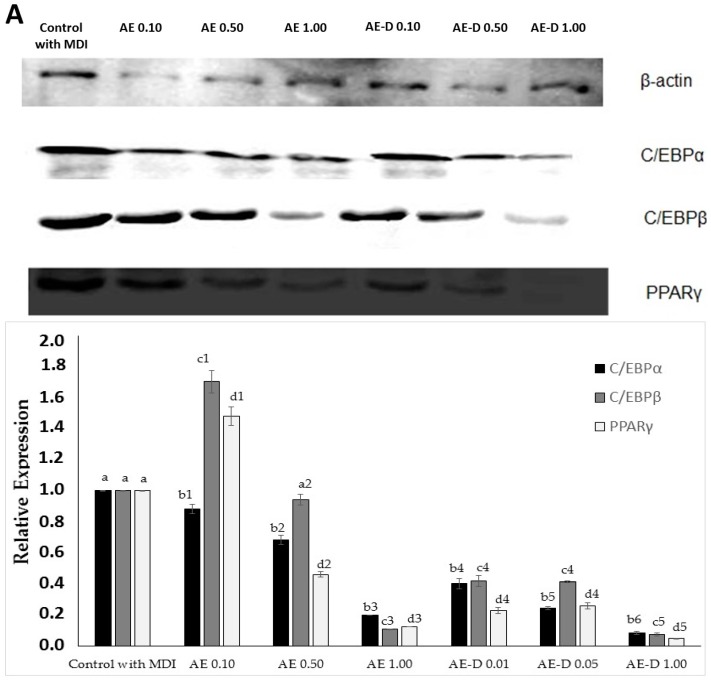
Effects of *Baccharis trimera* aqueous extract (AE) and AE with decoction (AE-D), both with adipogenic growth medium (MDI), on the expression of adipocyte markers. (**A**) Equal amounts of protein (50 µg) of cell lysate were analyzed by Western blotting to detect β-actin, CCAAT enhancer-binding protein α (C/EBPα), CCAAT enhancer-binding protein β (C/EBPβ), and gamma receptors by peroxisome proliferators (PPARγ) levels; (**B**) The graph of the protein relative expressions was obtained as described in materials and methods. Control, Control with MDI. Values are expressed as mean ± standard deviation (*n* = 3). Letters a,b,c,d indicate significant differences between different concentrations and the control. Numbers 1,2,3,4,5 indicate significant differences between each protein analyzed in the different concentrations between each extract as calculated by one-way analysis of variance (ANOVA) followed by the Student’s *t*-test (*p* < 0.05).

After 15 days of differentiation, the 3T3-L1 cells were lysed and the levels of C/EBPα, C/EBPβ, and PPARγ were measured via Western blotting analysis. As shown in Figure 5, expression of C/EBPβ and PPARγ significantly increased in the presence of 0.1 mg/mL of both extracts, while the level of C/EBPα was not affected.

When the concentration of the samples was increased from 0.1 to 0.5 mg/mL, in the presence of both extracts the levels of all proteins decreased. In addition, this decrease was more pronounced when the cells were treated with AE-D. In case of this extract, the expression of C/EBPα, C/EBPβ, and PPARγ decreased 75%, 78%, and 79%, respectively, compared to the levels observed when cells were exposed to 0.1 mg/mL extract.

The best inhibition levels were obtained when the cells were exposed to 1.0 mg/mL of each extract. In the presence of AE, the level of the three proteins decreased to below 80%. However, the effect of AE-D was more evident since it decreased the level of all three proteins by 90%.

### 2.7. High-Performance Liquid Chromatography with Diode Array Detection (HPLC-DAD) Profile of AE and AE-D Extracts from B. trimera

**Figure 6:**
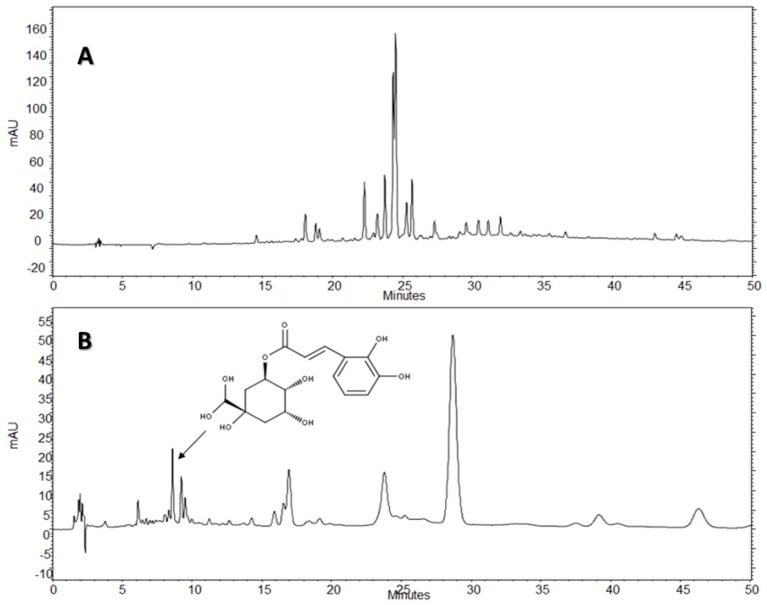
Chromatogram from a high performance liquid chromatography with diode array detector (HPLC-DAD) trial of the aqueous extract with decoction (AE-D) of *Baccharis trimera*: (**A**) Method 1; and (**B**) Method 2. SP: column C18 Phenomenex-Luna^®^ (250 × 4.6 mm, 5 µm); Mobile phase: acetonitrile gradient: acetic acid 0.3%; Detection: 340 nm. Chlorogenic acid was identified (arrow).

**Figure 7:**
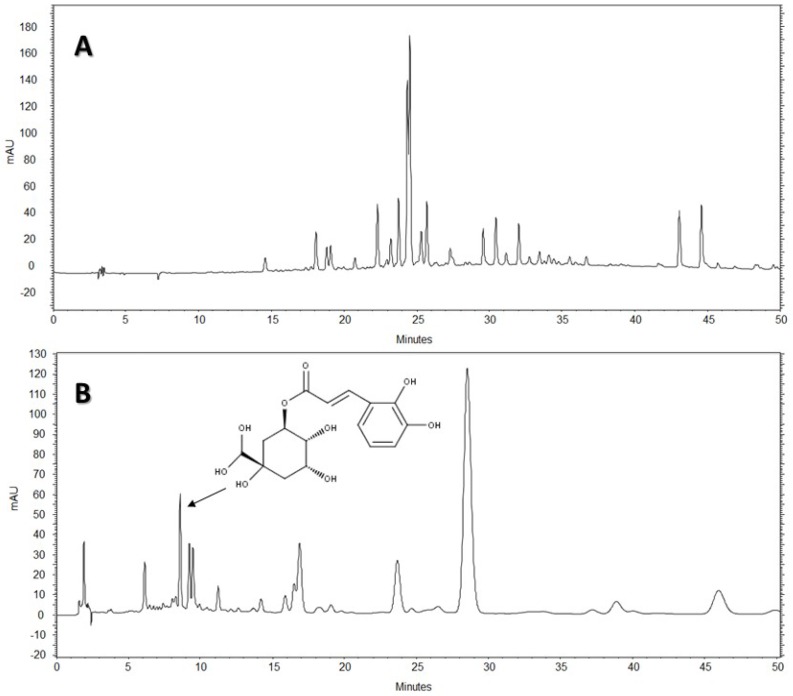
Results from high-performance liquid chromatography with diode array detector (HPLC-DAD) of the aqueous extract (AE) of *Baccharis trimera*: (**A**) Method 1; and (**B**) Method 2. SP: column C18 Phenomenex-Luna^®^ (250 × 4.6 mm, 5 µm); Mobile phase: acetonitrile gradient: acetic acid 0.3%; Detection: 340 nm. Chlorogenic acid was identified (arrow).

The chromatographic fingerprints of AE and AE-D, obtained using HPLC-DAD, are depicted in Figure 6 and Figure 7, respectively.

The chromatogram of the heated AE-D of *B. trimera* obtained using Methods 1 and 2 showed absorption characteristics similar to those of flavonoids and phenolic acids at the wavelengths analyzed, with at least 15 peaks in the chromatogram at a wavelength of 340 nm for Method 1 (see Materials and Methods), and 10 peaks for Method 2 (see Materials and Methods) (Figure 6A,B). Among the flavonoids, most had a UV spectrum similar to glycosylated flavonoids derived from apigenin (267 nm II band and 336 nm I band).

The chromatogram of the AE of *B. trimera* using Methods 1 and 2 showed absorption characteristics similar to those of flavonoids and phenolic acids at the wavelengths tested, with at least 17 peaks in the chromatogram at a wavelength of 340 nm using Method 1, and 13 peaks using Method 2 (Figure 7A,B). Therefore, in the AE extract, most flavonoids had a UV similar spectrum to glycosylated flavonoids derived from apigenin.

As shown in Figure 6 and Figure 7, there were significant differences between the chromatographic profiles presented by the AE-D and AE from *B. trimera*, demonstrating that the method used to obtain the extract can interfere with its qualitative and quantitative composition. In the chromatogram at 340 nm for both Methods 1 and 2, the extracts showed similar composition, differing mainly in the amount of the compounds. The AE had the largest compound content, especially in relation to the major peak.

Standards peaks were compared to the corresponding peaks in the extracts according to the tR and UV spectra, suggesting that chlorogenic acid (CGA) was the peak seen at 8.60 min and UV 325 nm in Method 1. Thereafter, by the analysis of standards and co-injection of extract + standard, it was possible to observe the increase in peak area for this standard analyzed. This suggests, once again, that CGA may be present in extracts.

## 3. Discussion

The presence of a large amount of reactive oxygen species (ROS) in preadipocyte cytoplasm is related to the acceleration of adipocyte differentiation through the expression of adipogenic genes in response to oxidative stress [18]. Furthermore, antioxidant and anti-adipogenic effects of several natural compounds are known to suppress adipogenesis by preventing increased ROS levels [8]. The decrease in excessive ROS levels and the inhibition of adipocyte differentiation are important strategies for preventing various chronic diseases related to oxidative stress and obesity. With this in mind, we first evaluated the antioxidant potential of *B. trimera* extracts.

ROS are formed through a chain reaction involving three steps (initiation, propagation, and termination). Thus, different methods were used to evaluate the antioxidant properties of AE, AE-D and ME at different stages: initiation (total antioxidant capacity (TAC) and reducing power), propagation (chelation of copper and iron), and termination (scavenging of the hydroxyl superoxide radical and of hydrogen peroxide), as shown in Figure 1.

In the TAC assay, our results were similar to those obtained by Oliveira et al. [19]. However, our work is the first to evaluate *B. trimera* extracts via a reducing power assay. Both of these tests evaluated the capacities of the samples to donate electrons and/or a hydrogen atom and break the free radical chain, however the test conditions are not similar. Therefore, when a compound acts as a donor in these two tests, it is an indication that it can act as an antioxidant in different conditions, such as in different cellular microenvironments.

All *B. trimera* extracts were able to scavenge superoxide radicals in a dose-dependent manner (Figure 1F). The superoxide radical is a highly toxic species produced by innumerable biological and photochemical reactions. This radical is a ROS that can damage various molecules, including proteins, lipids, and cellular DNA, causing cell and tissue damage as well as a number of diseases [20]. It is produced in vivo and leads to the formation of hydrogen peroxide (H_2_O_2_), another oxidant agent very harmful to cells, via a dismutation reaction. Thus, by scavenging superoxide radicals, the extracts also inhibit the formation of H_2_O_2_. 

In cells, H_2_O_2_ reacts with transition metal ions such as Cu^2+^ and Fe^2+^ to generate hydroxyl radicals via Fenton or Haber–Weiss reactions. The hydroxyl radical is the most reactive of ROS, causing serious damage to biomolecules. Its elimination is highly desirable since the elevated reactivity of this radical is associated with cellular damage, leading to several diseases [21]. In our study, all three extracts, particularly AE-D and ME, showed hydroxyl radical scavenging activity. Vieira and Co-workers [22] have also shown that aqueous and ethanol *B. trimera* extracts were able to scavenge hydroxyl radicals. They demonstrated that the aqueous extract was a stronger scavenger than the ethanol extract, which agrees with our data. However, these authors did not address whether the plant extracts only scavenge hydroxyl radicals or if they also act as a metal chelator. Our results show that the extracts have also metal chelating activity, especially copper chelating action, which also leads to a reduction in hydroxyl radical generation.

Still with respect to the chelating activity of the extracts. We can see in Figure 1, that all the extracts showed a much higher chelating activity of copper than the chelating activity of copper. Aqueous extracts of *B. trimera* have in their composition ruthin [11], apigenin, quercetin, and luteolin [23]. These phenolic compounds are agents with high copper chelating capacity and low iron chelating capacity [24,25]. What justifies the high copper chelating activity of aqueous extracts of *B. trimera.*

Overall, our data showed the great potential of the *B. trimera* extracts as antioxidant agents. The scavenging action of plant extracts has been correlated to the presence of phenolic compounds [26]. Indeed, we found large amount of these compounds in the extracts (Table 1). However, ME showed similar antioxidant activity to other extracts, although it contains fewer phenolic compounds. One possible explanation is that the different conditions of extraction allowed ME to contain large amounts of phenolic compounds that act as antioxidants. Another possibility is that the methanol extracted some compounds that were not extracted by water, and that these compounds are more powerful than those found in AE and AE-D. However, the confirmation of these hypotheses requires further investigations.

Transition metal ions including Cu^2+^ and Fe^2+^ can increase adipose tissue mass in rats [27]. Furthermore, since these metals increase the expression of adipogenic transcriptional factors, they contribute to 3T3-L1 cell differentiation. Excessive ROS generation is associated with increased lipid accumulation during adipocyte differentiation [28]. With this in mind, and after we identified the potent antioxidant activity of the extracts as both copper chelators and ROS scavengers, we decided to evaluate the anti-adipogenic activity of these extracts.

3T3-L1 cells have served as a well-documented model system used to develop anti-adipogenic agents. Therefore, it was first necessary to assess the effect of the extracts on the viability of these cells. Since ME showed anti-proliferative activity against 3T3-L1 cells (Figure 2), it was not used in next steps of this study. 

As shown in Figure 3A,B and Figure 4, the AE and AE-D were able to reduce the amount of neutral lipids within the cell, a fact evidenced by the reduced staining in the cells. In order to understand their effect, we also determined the level of the transcription factors C/EBPβ, PPARγ, and C/EBPα that play key roles in the complex transcriptional cascade of adipocyte differentiation. An increase in these factors in turn leads to increased levels of molecules involved in lipid synthesis such as fatty acid synthase, fatty acid binding protein, leptin, and adiponectin [29]. The results of the trial showed that C/EBPβ, PPARγ, and C/EBPα levels in the 3T3-L1 cells were reduced after treatment with AE and AE-D (Figure 5).

However, these data still need to be understood. The blotting analysis showed AE and AE-D (0.10 mg/mL) at low concentration stimulates the expression of PPARγ and C/EBPβ. However, an adipogenic effect was not observed (Figure 3 and Figure 4), probably because the level of C/EBPα are not high. C/EBPβ is known to be a central factor in adipocyte diffusion and regulates C/EBPα levels [4,5]. Thus, the data obtained here lead us to propose that there are one or more compounds in the extracts that prevent the action of C/EBPβ on C/EBPα. However, the confirmation of these hypotheses requires further investigations.

*B. trimera* extracts have been identified as anti-obesity agents [13,14,15]. However, to our knowledge, there are no other groups that have evaluated the effect of *B. trimera* extracts as anti-adipogenic agents, which made a comparison of our results with those from other research groups impossible. Nevertheless, based on our data, we can say that the extracts are anti-adipogenic agents.

The HPLC-DAD analysis showed that AE-D and AE extracts contain several different types of phenolic compounds (Figure 6 and Figure 7). This result is consistent with those of previous reports. In fact, phytochemical analyses of *B. trimera* extracts showed the presence of several phenolic compounds such as rutin [11], eupafolin, hispidulin, apigenin, quercetin, and luteolin, [23], of which these last three and rutin are possible anti-obesity agents [9]. In our study, the data suggest the presence of CGA in *B. trimera* extract composition. CGA acts as donor of hydrogen atoms to reduce free radicals and to inhibit oxidation reactions, becoming oxidized to its phenoxyl radical that is quickly stabilized by resonance stabilization as a result [30]. Arçari and co-workers [31] showed that CGA is a more potent anti-adipogenic compound than rutin and quercetin, since even after using a tenfold amount of these molecules, they failed to achieve the same degree of adipogenesis inhibition that they obtained using CGA. Thus, we suggested that CGA is another molecule responsible for the anti-adipogenic activity of *B. trimera* extracts.

Before the all data, a question arose. Why was EA-D a more potent anti-adipogenic agent than AE? The HPLC-DAD analysis showed similar composition of these extracts, differing mainly in the concentration of the compounds. In fact, the AE extract showed the highest content of active compounds. However, AE-D was more powerful than AE in three different antioxidant tests (TAC, reducing power, and hydroxyl radical scavenging). Thus, we do not rule out the possibility that additional compound(s) present in the AE-D that are not found in the AE might also show anti-adipogenic activity. The confirmation of this hypothesis requires further investigations.

## 4. Materials and Methods

### 4.1. Materials

Potassium ferricyanide, ferrous sulfate (II), ethylenediaminetetraacetic acid (EDTA), gallic acid, ammonium molybdate, hydrogen peroxide 30%, acetic acid, Folin–Ciocalteu phenol reagent, ethanol, and sulfuric acid were obtained from Merck (Darmstadt, Germany). Nitro blue tetrazolium (NBT), monosaccharides, EDTA, ascorbic acid, methionine, bovine serum albumin (BSA), pyrocatechol violet, ascorbic acid, and ammonium molybdate were purchased from Sigma-Aldrich Co. (St. Louis, MO, USA). Sodium bicarbonate, culture media components (Minimum essential Dulbecco’s modified Eagle medium (DMEM)), non-essential amino acids, fetal bovine serum, sodium pyruvate, and phosphate buffered saline (PBS) were purchased from Invitrogen Canada Inc. (Burlington, ON, Canada). Acetonitrile (HPLC grade) purchased from Panreac^®^ (São Paulo, Brazil). Acetic acid was provided by Vetec^®^ (São Paulo, Brazil). Water was purified with a Milli-Q system (Millipore^®^, Bedford, MA, USA). All other solvents and chemicals were of analytical grade.

### 4.2. Acquisition of B. trimera Extracts

*B. trimera* plants were collected in May and June 2012 in Brasilia, DF, Brazil. A specimen (No. 15694) of the plant was deposited in the Herbarium at the Department of Botany, “Universidade Federal do Rio Grande do Norte” (UFRN), Natal, RN, Brazil. The selected shoots were dehydrated in an aerated oven at 45 °C for three days and then crushed. From the ground material three different extracts were prepared according as described above.

Approximately 100 g of crushed material was boiled in 250 mL of water for 10 min and filtered, concentrated under reduced pressure at −80 °C, and lyophilized. The sample obtained was named AE-D.

ME was obtained after ground material (~100 g) was macerated into methanol (250 mL) for 24 h, at room temperature (~22 °C), under stirring for 24 h (three replicates). The mixture was then filtered, concentrated under reduced pressure at −80 °C, and lyophilized.

In order to obtain the aqueous extract (AE), the ground material (~100 g) was macerated into 250 mL of distillated water at room temperature under stirring for 24 h. The mixture was then filtered, concentrated under reduced pressure at −80 °C, and lyophilized.

### 4.3. Chemical Analysis 

Total sugar, protein, and phenolic compound contents were determined as described previously [32]. Phenolic compounds were determinate via the Folin-Ciocalteu phenol reagent method using gallic acid as a standard. The amounts of protein were measured using the Coomassie Brilliant Blue reagent, with BSA as a standard. Total amounts of carbohydrates were determined using the phenol-H_2_SO_4_ method with d-glucose as a standard.

### 4.4. HPLC-DAD Profile of AE and AE-D Extracts from the Leaves of B. trimera

HPLC-DAD analyses were performed using an HPLC Merck-Hitach^®^ (Hichrom model) instrument equipped with a diode array detector, quaternary pump, oven column, and autoinjector. For the analysis of the compounds, two methods were performed using a Phenomenex^®^ Luna RP-18 column (250 × 4.6 mm, 5 µm particle size) and the eluents were: (A) acetic acid 0.3% and (B) acetonitrile. The following gradient (*v*/*v*) was applied: 0–50% B, 0–50 min for Method 1, and 5–10% B, 0–5 min; 10% B, 5–50 min for Method 2. Both methods had a total analysis time of 50 min. Flow elution was 1.0 mL/min for Method 1 and 1.5 mL/min for Method 2, and the injection volume was 20 µL for both methods. Detection was performed at 254, 280, and 340 nm, with the acquisition of UV spectra in the range of 200 to 400 nm.

The AE of *B. trimera* (heated and unheated) was compared against standards of CGA (≥95%), ellagic acid (≥95%), gallic acid (≥95%), vitexin (≥95%), all isovitexin (≥98%), all acquired from Sigma-Aldrich^®^. The AE of *B. trimera* and standards were mixed in methanol:water, 1:1 (*v*/*v*). The final concentration of the extract was 3.0 mg/mL. For standards, the final concentration was 100 μg/mL. In this technique, the identification of compounds was based on a comparison of retention times, UV spectra of the major peaks, and the increase in peak area after co-injection of standards and extracts. Each trial was performed in triplicate. After total dissolution and prior to analysis, the samples and solvents were filtered through a 0.45 μm membrane (MillexTM, Merck^®^).

### 4.5. Antioxidant Activity

The antioxidant activities of *B. trimera* extracts were measured using the methods described by Melo et al. (2014) [33], and/or Melo-Silveira et al. (2014) [34].

#### 4.5.1. Determination of Total Antioxidant Capacity

The assay used was based on the reduction of Mo^6+^ to Mo^5+^ by the samples and the subsequent formation of a green phosphate-molybdate complex at an acidic pH. Tubes containing extracts and reagent solution (0.6 M sulfuric acid, 28 mM sodium phosphate, and 4 mM ammonium molybdate) were incubated at 95 °C for 90 min. After the mixture had cooled to room temperature, the absorbance of each solution was measured at 695 nm against a blank. The TAC was measured in ascorbic acid mg/extract g, termed equivalent of ascorbic acid.

#### 4.5.2. Reducing Power

The reducing power assay depends on the reduction of the potassium ferricyanide by the samples. Briefly, the *B. trimera* extracts were mixed with a phosphate buffer 0.2 M (pH 6.6), potassium ferricyanide (1% *m*/*v*) and incubated at 50 °C for 20 min. One addition of trichloroacetic acid (10% *m*/*v*) was used to in order to stop the reaction. Distilled water and ferrous chloride (0.1% *m*/*v*) were added to the solution and the absorbing capacities were measured at 700 nm. Results were calculated as an activity percentage, considering the largest concentration of ascorbic acid (the standard) as 100% activity.

#### 4.5.3. Ferric Chelating

The chelating ability of samples was evaluated as described below: Each extract at different concentrations was added to the reaction mixture containing FeCl_2_ (0.05 mL, 2 mM) and ferrozine (0.2 mL, 5 mM). The mixture was shaken and incubated for 10 min at room temperature and absorbance of the mixture was measured (562 nm) against a blank. EDTA was used as positive control.

The chelating effect was calculated using the corresponding absorbance (A) in the formula given below, where control is the absorbance in the absence of chelating agents:

(1) $$\mathrm{Chelating}\;\mathrm{Effect}\;\left(\%\right)=\left(\frac{A_{\mathrm{control}}-A_{\mathrm{sample}}}{A_{\mathrm{control}}}\right)\times 100$$

#### 4.5.4. Copper Chelation

The test was carried out in 96-well microplates with 0.2 mL of reaction mixture containing varying amount of *B. trimera* extracts, pyrocatechol violet (4 mM), and copper II sulfate pentahydrate (50 mg/mL). All wells were stirred and the solution absorbance was measured at 632 nm. The control group was obtained using the same reagents in the absence of chelating agents. The ability of the *B. trimera* extracts to chelate the copper ion was determinate using the following formula:

(2) $$\mathrm{Copper}\;\mathrm{chelation}\;\left(\%\right)=\left(\frac{A_{\mathrm{control}}-A_{\mathrm{sample}}}{A_{\mathrm{control}}}\right)\times 100$$

#### 4.5.5. Hydroxyl Radical Scavenging Activity Assay

The Fenton’s reaction (Fe^2+^ + H_2_O_2_→Fe^3+^ + OH^−^ + OH˙) was used in order to determinate the scavenging activity of *B. trimera* extracts against the hydroxyl radical. These data were expressed as inhibition rates. Hydroxyl radicals were generated mixing 3 mL sodium phosphate buffer (150 mM, pH 7.4), 10 mM FeSO_4_·7H_2_O, 10 mM EDTA, 2 mM sodium salicylate, and 30% H_2_O_2_ (200 mL) and varying extract concentrations. In the control, sodium phosphate buffer was replaced by H_2_O_2_. The solutions were incubated at 37 °C for 1 h, and the presence of OH^−^ was detected by monitoring absorbance at 510 nm. Gallic acid was used as the positive control.

#### 4.5.6. Superoxide Radical Scavenging Activity Assay

This test depends on the ability of samples to inhibit the photochemical reduction of nitroblue tetrazolium (NBT) in the riboflavin–light–NBT system. Each 3 mL of reaction mixture contained 75 mM NBT, 50 mM phosphate buffer (pH 7.8), 2 mM riboflavin, 13 mM methionine, 100 mM EDTA, and 1 mL sample solution. After the production of blue formazan the increase in absorbance at 560 nm after 10-min illumination from a fluorescent lamp was determined. The entire reaction assembly was enclosed in a box lined with aluminum foil. An identical reaction was maintained in the dark and used as a blank. The positive control was the Gallic acid. Results were accounted in scavenging percentage.

### 4.6. Cell Culture, Differentiation and BrdU Assay

3T3-L1 preadipocyte cells were purchased from a cell bank in Rio de Janeiro, RJ, Brazil (CR089-BCRJ/UFRJ) and maintained with 10% FBS/DMEM containing 4.5 g/L glucose, 100 U/mL penicillin, 0.1 mg/mL streptomycin, and 0.25 mg/mL amphotericin B at 37 °C in 5% CO_2_ incubator. Confluent cells were differentiated by incubation with the hormone mixture MDI containing 10 µg/mL insulin, 1 μM dexamethasone, and 0.5 mM IBMX, in 10% FBS/DMEM for 72 h. Thereafter, the cells were maintained in post-differentiation medium containing 10 g/mL insulin in 10% FBS/DMEM in the presence of various concentrations (0.05; 0.10; 0.25; 0.50 and 1.00 mg/mL) of *B. trimera* extracts and the medium was replaced every 3 days. The same concentration of *B. trimera* extract was supplemented at 3-day intervals when culture medium was replaced. Differentiation, as measured by the expression of adipogenic markers and appearance of lipid droplets, was completed at Day 15 as measured with the use of the dye Oil Red O.

Anti-proliferative testing was conducted using 5-Bromo-2-Deoxyuridine (BrdU) assay, as described by Nobre et al. 2014 [35]. 3T3-L1 cells were seeded into 96-well plates at a density of 5 × 10^3^ cells/well and allowed to attach overnight in 250 μL of fresh medium at culture conditions (24 h, at 37 °C and 2.5% CO_2_). After 12 h, the medium was removed and the extracts B in medium was added to a final concentration of 0.05–1.0 mg/mL and incubated for 24 or 48 h. After incubation, traces of extracts were removed by washing the cells twice with 200 μL PBS and the BrdU incorporation was determined according to the manufacturer’s instruction (BrdU cell proliferation assay kit-Cell Signaling, Danvers, MA, USA).

### 4.7. Oil Red O Staining and Free Glycerol

At day 15 after the induction of differentiation, cells were stained with Oil Red O. Cells were washed twice with PBS and fixed with 3.7% formaldehyde for 10 min. Fixed cells were stained with 0.2% Oil Red O-isopropanol for 1 h and the excess of stain was washed by 70% ethanol and water. Cells were then photographed using microscope (Nikon EclypseTi-E). Stained oil droplets was dissolved with isopropanol and quantified by spectrophotometric analysis at 510 nm. The optical density in only-MDI-treated cells was taken as 100% of relative lipid content. Results were represented as relative lipid contents of each experimental group. 

To determine free glycerol, we used a Sigma kit (Sigma-Aldrich Co., St. Louis, MO, USA). The free glycerol reagent was made up according to the manufacturer’s instructions at room temperature. For the test were prepared solution without samples (10 µL water + 400 µL reagent), the standard (5 µL glycerol + 400 µL reagent) and samples (10 µL sample + 400 µL reagent). To complete mixing of the components, shaken by inversion and led to heating in a water bath at 37 °C for 5 min. The absorbance of the mixture was measured at 540 nm for the quantitative enzymatic determination of glycerol. The blank was used as negative control and the standard as positive. The procedure involves enzymatic hydrolysis by lipase of the triglycerides to glycerol and free fatty acids. The increase in absorbance at 540 nm is directly proportional to the free glycerol concentration of the sample.

### 4.8. Western Blotting Analysis

3T3-L1 cells were plated in 100 mm plates and stimulated to differentiate into adipocytes (as described above) in the presence or absence of the sample. After 15 days of differentiation, cells were removed from the plates (two plates per treatment) and subjected to washing in ice cold PBS and centrifugation. To obtain the total cellular protein extract, the cells were re-suspended in lysis buffer (50 mM Tris-HCl (pH 7.4); 1% Tween 20; 0.25% sodium deoxycholate; 150 mM NaCl; 1 mM EGTA; 1 mM Na_3_VO_4_; 1 mM NaF; and protease inhibitors) for 2 h on ice.

After removal of cellular debris by centrifugation, the protein concentration of the extracts was determined using Bradford reagent (Bio-Rad, Hercules, CA, USA). The extracts were re-suspended in sample buffer (100 mM Tris-HCl (pH 6.8), 200 mM dithiothreitol, 4% sodium dodecyl sulfate (SDS), 0.1% bromophenol blue and 20% glycerol), and these solutions were boiled for 5 min. For each sample, approximately 30 μg of protein was subjected to SDS-polyacrylamide gel electrophoresis (PAGE) with subsequent transfer to polyvinylidene fluoride (PVDF) membranes (Millipore, Bedford, MA, USA). The membranes were blocked by a solution containing nonfat milk (1%) or albumin (1%) in tris-buffered saline (0.05%) and Tween 20 (TBST buffer) and incubated 12 h at 4 °C with appropriate primary antibody at a dilution of 1:1000. After further washing in TBST buffer, the membranes were incubated with appropriate secondary antibody (anti-mouse or anti-rabbit) conjugate with peroxidase (dilution 1: 1000) in blocking buffer for 1 h. Detection was performed using chemiluminescence.

The bands in the gel were quantified by densitometry and a ratio between the values obtained for the C/EBPα, C/EBPβ, and PPARγ proteins and the value obtained for actin was used to construct Figure 5.

The primary antibodies used specific for β-actin (sc-130656) were purchased from Santa Cruz Biotechnology, Inc. (Santa Cruz, CA, USA); C/EBPα (8178), C/EBPβ (3087), and PPARγ (2443) were obtained from Cell Signaling Technology (Beverly, MA, USA). The horseradish peroxidase (HRP) Secondary antibodies were also obtained from Cell Signaling Technology (Beverly, MA, USA).

### 4.9. Statistical Analysis

All experiments were repeated at least three times, and all values are given as mean ± standard deviation (S.D.) of triplicates. One-way analysis of variance (ANOVA) followed by a Student’s *t*-test was used to analyze statistical significance. Results with *p* < 0.05 were considered as statistically significant.

## 5. Conclusions

This report suggests that *B. trimera* extracts have antioxidant activity, prevent many diseases caused by oxidative stress, and can attenuate adipogenesis during the adipogenic differentiation process, since they were anti-adipogenic and antioxidant agents. The beneficial role of *B. trimera* on obesity is connected to its antioxidant actions. *B. trimera* extracts, especially AE-D, regulate adipogenesis by decreasing the levels of the adipogenic transcription factors C/EBPα, C/EBPβ, and PPARγ. In addition, we suggested the presence of CGA in *B. trimera,* and it may be another molecule responsible for in the *B. trimera* anti-adipogenic effect. This is the first study to demonstrate the potential effect of *B. trimera* in the differentiation of preadipocyte 3T3-L1 into adipocytes. Thus, we suggest that *B. trimera* shows promise as an alternative to available therapeutic strategies for obesity. However, more studies are needed to understand and clarify the mechanisms of action of *B. trimera* in weight loss.

## Figures and Tables

**Table 1:** The amount of sugar, protein and phenolic compounds in *Baccharis trimera* extracts.

Compounds	AE	AE-D	ME
Sugar (mg)	18.4 ± 0.84 ^a^	23.7 ± 0.23 ^a^	14.1 ± 0.19 ^b^
Proteins (mg)	5.2 ± 0.15 ^a^	5.8 ± 1.21 ^a^	8.8 ± 0.97 ^b^
Phenolic (mg)	25.8 ± 1.28 ^a^	29.4 ± 0.64 ^a^	20.7 ± 0.66 ^b^

AE, aqueous extract; AE-D, aqueous extract from decoction; ME, methanol extract. Letters a,b represent a significant difference between the samples by the one-way analysis of variance analyses (ANOVA) followed by the Student’s *t*-test (*p* < 0.05).
